# Identification of Genes Required for Glucan Exopolysaccharide Production in Lactobacillus johnsonii Suggests a Novel Biosynthesis Mechanism

**DOI:** 10.1128/AEM.02808-19

**Published:** 2020-04-01

**Authors:** Melinda J. Mayer, Alfonsina D’Amato, Ian J. Colquhoun, Gwénaëlle Le Gall, Arjan Narbad

**Affiliations:** a Gut Microbes and Health Institute Strategic Programme, Quadram Institute Bioscience, Norwich, United Kingdom; b Analytical Sciences Unit, Quadram Institute Bioscience, Norwich, United Kingdom; c Department of Medicine, Faculty of Medicine and Health Sciences, University of East Anglia, Norwich, United Kingdom; University of Illinois at Urbana-Champaign

**Keywords:** exopolysaccharide, alpha glucan, *Lactobacillus johnsonii*, proteomics, glycosyltransferase, nuclear magnetic resonance

## Abstract

Lactobacillus johnsonii FI9785 makes two capsular exopolysaccharides—a heteropolysaccharide (EPS2) encoded by the *eps* operon and a branched glucan homopolysaccharide (EPS1). The homopolysaccharide is synthesized in the absence of sucrose, and there are no typical glucansucrase genes in the genome. Quantitative proteomics was used to compare the wild type to a mutant where EPS production was reduced to attempt to identify proteins associated with EPS1 biosynthesis. A putative bactoprenol glycosyltransferase, FI9785_242 (242), was less abundant in the Δ*eps_cluster* mutant strain than in the wild type. Nuclear magnetic resonance (NMR) analysis of isolated EPS showed that deletion of the *FI9785_242* gene (*242*) prevented the accumulation of EPS1, without affecting EPS2 synthesis, while plasmid complementation restored EPS1 production. The deletion of *242* also produced a slow-growth phenotype, which could be rescued by complementation. 242 shows amino acid homology to bactoprenol glycosyltransferase GtrB, involved in O-antigen glycosylation, while *in silico* analysis of the neighboring gene *241* suggested that it encodes a putative flippase with homology to the GtrA superfamily. Deletion of *241* also prevented production of EPS1 and again caused a slow-growth phenotype, while plasmid complementation reinstated EPS1 synthesis. Both genes are highly conserved in L. johnsonii strains isolated from different environments. These results suggest that there may be a novel mechanism for homopolysaccharide synthesis in the Gram-positive L. johnsonii.

**IMPORTANCE** Exopolysaccharides are key components of the surfaces of their bacterial producers, contributing to protection, microbial and host interactions, and even virulence. They also have significant applications in industry, and understanding their biosynthetic mechanisms may allow improved production of novel and valuable polymers. Four categories of bacterial exopolysaccharide biosynthesis have been described in detail, but novel enzymes and glycosylation mechanisms are still being described. Our findings that a putative bactoprenol glycosyltransferase and flippase are essential to homopolysaccharide biosynthesis in Lactobacillus johnsonii FI9785 indicate that there may be an alternative mechanism of glucan biosynthesis to the glucansucrase pathway. Disturbance of this synthesis leads to a slow-growth phenotype. Further elucidation of this biosynthesis may give insight into exopolysaccharide production and its impact on the bacterial cell.

## Abstract (precis)

Exopolysaccharides are key components of the surfaces of their bacterial producers, contributing to protection, microbial and host interactions, and even virulence. They also have significant applications in industry, and understanding their biosynthetic mechanisms may allow improved production of novel and valuable polymers. Four categories of bacterial exopolysaccharide biosynthesis have been described in detail, but novel enzymes and glycosylation mechanisms are still being described. Our findings that a putative bactoprenol glycosyltransferase and flippase are essential to homopolysaccharide biosynthesis in Lactobacillus johnsonii FI9785 indicate that there may be an alternative mechanism of glucan biosynthesis to the glucansucrase pathway. Disturbance of this synthesis leads to a slow-growth phenotype. Further elucidation of this biosynthesis may give insight into exopolysaccharide production and its impact on the bacterial cell.

## INTRODUCTION

Production of exopolysaccharides (EPS) has a large impact on the nature of the bacterial surface and hence on interactions with the environment, hosts and host defense systems, and other microbes (1, 2). EPS can protect bacteria against environmental conditions, both outside and inside the host (1, 3, 4), and in the case of pathogens such as Streptococcus pneumoniae, they can have an important association with immune evasion and virulence (5). EPS can have immunomodulatory and protective properties in the host (6–9) and can affect the composition and function of the gut microbiota (10, 11). EPS can also play a crucial role in biofilm formation, adhesion to host cells, and colonization (3, 12–15). In addition to their biological importance, bacterial EPS have a range of technological applications in food, pharmaceutical, and other industries and may also have potential health benefits due to their activities in immune stimulation, antitumor activity, and lowering of blood cholesterol or as prebiotics (1, 2, 16, 17).

Lactobacillus johnsonii FI9785 is a poultry isolate which has shown promise as a competitive exclusion agent against Clostridium perfringens (18) and Campylobacter jejuni (19). This strain makes 2 capsular exopolysaccharides—EPS2, a heteropolysaccharide containing glucose and galactose encoded by a 14-gene *eps* operon of the Wzx/Wzy type, and EPS1, a branched dextran homopolysaccharide with an α-(1→6) backbone and α-(1→2) branches which are present on every unit of the backbone and consist of a single glucose (Glc) residue (20, 21). This is an unusual structure which has not been described in other bacteria, although a small percentage of α-(1→2) branches were seen in dextran produced by Leuconostoc citreum E497 (22). Glucansucrases have been shown to synthesize homopolysaccharides in lactic acid bacteria, using sucrose as a substrate (17). However, L. johnsonii FI9785 makes EPS1 in the absence of sucrose, and there is no glucansucrase gene present in the genome, suggesting a different mode of biosynthesis (20). In previous work, the 14-gene *eps* operon (loci *FI9785_1170* to *FI9785_1183* inclusive, now renamed *FI9785_RS05260* to *FI9785_RS05325*) was removed by deletion mutagenesis to create the mutant strain Δ*eps_cluster* (20), and a second mutant strain where just the transcriptional regulator *epsA* (*FI9785_1183*) was deleted was also constructed (23). Although these mutations were expected to just affect the synthesis of EPS2 and not EPS1, these strains did not show an EPS layer by transmission electron microscopy (TEM), and nuclear magnetic resonance (NMR) analysis of EPS extractions failed to identify either EPS1 or EPS2 (20, 23, 24). In this work, we compared the proteome of the wild-type L. johnsonii FI9785 EPS producer with the Δ*eps_cluster* mutant strain to attempt to identify proteins involved in homopolysaccharide biosynthesis.

## RESULTS

### Comparative quantitative proteomic analyses identified proteins affected by deletion of the *eps* cluster.

**FIG 1:**
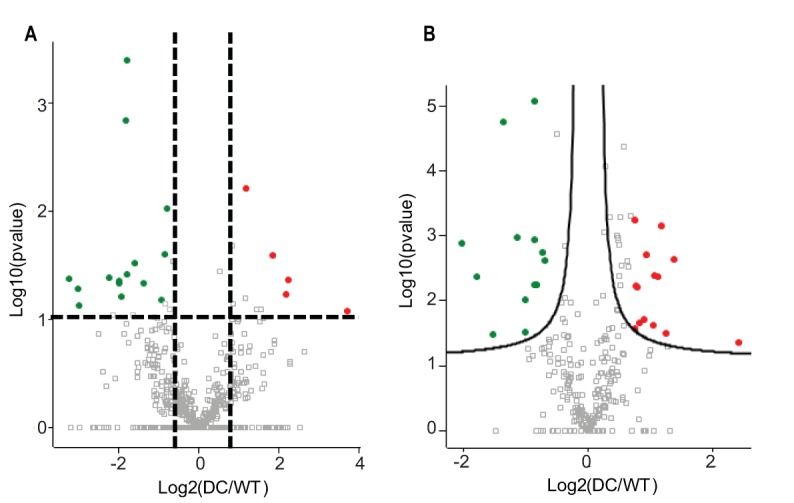
Volcano plots of differentially expressed proteins. Results compare L. johnsonii Δ*eps_cluster* (DC) versus FI9785 (WT) obtained using a two-sided *t* test in panels A (iTRAQ) and B (label-free experiments). Red indicates abundance higher in DC than WT; green indicates abundance lower in DC than WT (using a *P* value of less than 0.05).

**FIG 2:**
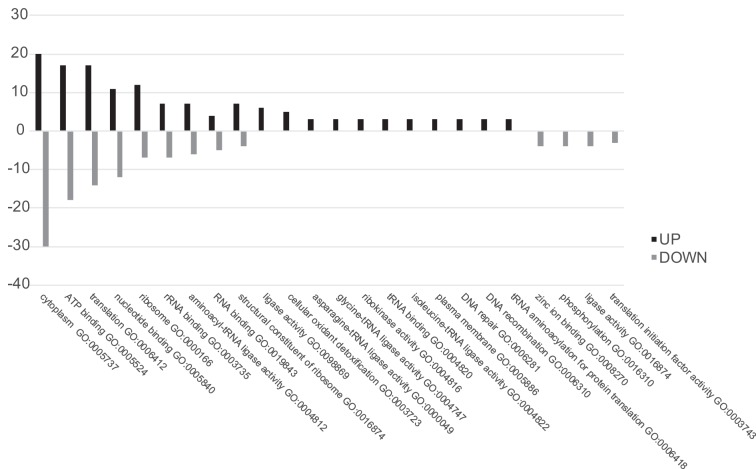
Gene Ontology analyses of differentially expressed proteins. On the *x* axis, the Gene Ontology enriched terms are shown, and on the *y* axis, the percentage of enrichment is shown. Up, processes enriched in the Δ*eps_cluster* mutant strain compared to the WT; Down, processes enriched in the WT compared to the mutant.

In order to identify proteins involved in EPS biosynthesis, the proteome of the wild type was compared to that of a mutant with a reduced EPS capsule to highlight proteins which were missing or downregulated in the mutant. Proteomic analysis of the soluble fractions of L. johnsonii FI9785 and the Δ*eps_cluster* strain identified several proteins which were differently expressed between the two strains. The protein samples were trypsin digested and labeled by iTRAQ (isobaric tag for relative and absolute quantitation) reagents, mixed and analyzed using nano-liquid chromatography tandem mass spectroscopy (nLC MS/MS) or directly analyzed without labeling for the label-free experiment. Andromeda analyses resulted in the identification of 699 soluble proteins (see Data Set S1 in the supplemental material), 49 of which were differentially expressed in the Δ*eps_cluster* strain versus the wild type (WT; Table 1). The volcano plots in Fig. 1 show the proteins which changed in abundance, obtained in iTRAQ (Fig. 1A) and label-free (Fig. 1B) experiments. The two different quantitative approaches allowed the quantitation of identical proteins with a similar ratio in the mutant and the control, e.g., D0R1R2, supporting the accuracy of the analyses, but also identified different proteins, allowing an in-depth characterization of proteins altered in the Δ*eps_cluster* strain. A total of 20 proteins were found at a higher level in the Δ*eps_cluster* strain, 4 identified by iTRAQ and 17 by the label-free approach, with only one found by both methods; the remaining 29 proteins were at higher levels in the WT, 17 found by iTRAQ and 14 by the label-free method, with 2 proteins identified by both methods (Table 1). In Fig. 2, enriched Gene Ontology (GO) terms of proteins found at different levels in the L. johnsonii FI9785 and Δ*eps_cluster* strains are described. Soluble proteins, mainly present in the cytoplasm, are involved in ATP binding (GO:0005524), translation (GO:0006412), nucleotide binding (GO:0000166), and transferase activity (GO:0016740) in the mutant strain. Almost half of the proteins with altered abundance were associated with ribosomal structure, translation, and protein biosynthesis, but some were more and some less abundant in the Δ*eps_cluster* strain, with no discernible pattern. No other biological processes seemed to be strongly impacted in the Δ*eps_cluster* strain. Although EPS is known to protect the cells from stress, there were no notable changes in stress response except a higher level of thiol peroxidase, commonly involved in cell redox homeostasis (Table 1).

**TABLE 1:** Quantified Lactobacillus johnsonii proteins using MaxQuant software in iTRAQ and label-free experiments^*a*^

Protein accession no.	Protein name	Gene name	No. of razor and unique peptides	Mol wt (kDa)	Score	log_2_ (D/WT)	D/WT ratio	WT/D ratio	iTRAQ^*b*^	Label free^*b*^	GO biological process
D0R1P2	Uncharacterized protein	*FI9785_401*	4	22	234	3.71	13.12	0.08	X		−
D0R498	Thiol peroxidase	*tpx*	3	18	29	2.41	5.30	0.19		X	Cell redox homeostasis; oxidation/reduction process; cellular oxidant detoxification
D0R5C5	Ribosomal silencing factor RsfS	*rsfS*	3	14	111	2.22	4.67	0.21	X		Mature ribosome assembly; negative regulation of ribosome biogenesis; negative regulation of translation; regulation of translation
D0R3E4	50S ribosomal protein L28	*rpmB*	3	7	26	2.17	4.49	0.22	X		Translation
D0R3T4	Aspartate-tRNA ligase	*aspS*	26	71	155	1.38	2.60	0.39		X	Translation; tRNA aminoacylation for protein translation; aspartyl-tRNA aminoacylation
D0R3V1	Glycine-tRNA ligase beta subunit	*glyS*	16	78	150	1.23	2.35	0.43		X	Translation; arginyl tRNA aminoacylation; glycyl tRNA aminoacylation
D0R277	Uncharacterized protein	*FI9785_219*	7	17	87	1.16	2.24	0.45		X	−
D0R5K0	Aspartyl/glutamyl-tRNA (Asn/Gln) amidotransferase subunit C	*gatC*	3	12	42	1.12	2.17	0.46		X	Translation; regulation of translational fidelity
D0R6B7	Deoxynucleoside kinase	*dgk1*	10	25	60	1.05	2.07	0.48		X	Nucleobase-containing compound metabolic process; deoxyribonucleoside monophosphate biosynthetic process; nucleotide biosynthetic process; phosphorylation
D0R362	Ribokinase	*rbsK*	16	33	146	1.04	2.05	0.49		X	Carbohydrate metabolic process; d-ribose metabolic process; phosphorylation; d-ribose catabolic process; carbohydrate phosphorylation
D0R1J3	30S ribosomal protein S12	*rpsL*	9	15	134	0.93	1.90	0.53	X	X	Translation
D0R2C4	Recombination protein RecR	*recR*	2	22	44	0.89	1.85	0.54		X	DNA repair; DNA recombination; cellular response to DNA damage stimulus
D0R4S3	Isoleucine-tRNA ligase	*ileS*	16	107	100	0.81	1.75	0.57		X	Translation; tRNA aminoacylation for protein translation; isoleucyl tRNA aminoacylation; aminoacyl-tRNA metabolism involved in translational fidelity
D0R1L4	30S ribosomal protein S5	*rpsE*	15	19	278	0.77	1.71	0.59		X	Translation
D0R5T0	Uncharacterized protein	*FI9785_1588*	10	21	53	0.76	1.70	0.59		X	−
D0R434	Asparagine-tRNA ligase	*asnS*	18	50	72	0.74	1.67	0.60		X	Translation; tRNA aminoacylation for protein translation; asparagyl tRNA aminoacylation
D0R3G2	30S ribosomal protein S16	*rpsP*	5	11	227	0.73	1.66	0.60		X	Translation
D0R5D2	50S ribosomal protein L35	*rpmL*	7	8	80	0.69	1.61	0.62		X	Translation
D0R4W9	ATP synthase subunit b	*atpF*	7	18	42	0.65	1.57	0.64		X	ATP biosynthetic process; ion transport; ATP synthesis coupled proton transport; ATP hydrolysis coupled cation transmembrane transport
D0R5D1	50S ribosomal protein L20	*rplT*	8	13	101	0.63	1.55	0.65		X	Ribosomal large subunit assembly; translation
D0R608	Chromosome partitioning protein ParB	*parB*	6	33	51	−3.26	0.10	9.60	X		
D0R4H4	Pseudouridine synthase	*FI9785_1123*	5	27	115	−3.02	0.12	8.09	X		Psuedouridine synthesis; RNA modification
D0R5U7	Elongation factor P	*efp*	8	21	105	−2.99	0.13	7.96	X		Translation; translational elongation; peptide biosynthetic process
D0R1R2	Putative glycosyl transferase	*FI9785_242*	8	35	46	−2.50	0.18	5.64	X	X	−
D0R254	Extracellular solute-binding protein PhnD	*phnD*	4	34	86	−2.26	0.21	4.78	X		Transmembrane transport
D0R5M6	Aggregation promoting factor	*apf2*	3	33	190	−2.04	0.24	4.12		X	−
D0R1L2	50S ribosomal protein L6	*rplF*	12	19	205	−2.01	0.25	4.04	X		Translation
D0R588	Peptide chain release factor 3	*prfC*	10	59	63	−1.99	0.25	3.97	X		Translation; translational termination; regulation of translational termination
D0R5Z4	Tagatose-6-phosphate kinase	*fruB*	4	33	153	−1.96	0.26	3.88	X		Carbohydrate metabolic process; lactose metabolic process; phosphorylation; carbohydrate phosphorylation
D0R4W2	MreB-like protein	*mbl*	8	35	318	−1.83	0.28	3.56	X		Cell morphogenesis
D0R4I9	Ribonuclease Z	*rnZ*	4	35	17	−1.82	0.28	3.52	X		tRNA processing; tRNA 3′-trailer cleavage, endonucleolytic; tRNA 3′-trailer cleavage; RNA phosphodiester bond hydrolysis, endonucleolytic
D0R1L5	50S ribosomal protein L30	*rpmD*	2	6	37	−1.81	0.29	3.50	X	X	Translation
D0R1U3	Tryptophan-tRNA ligase	*trpS*	8	39	269	−1.80	0.29	3.47	X		Translation; tRNA aminoacylation for protein translation; tryptophanyl tRNA aminoacylation
D0R5K3	ATP-dependent DNA helicase	*pcrA*	18	84	294	−1.60	0.33	3.04	X		DNA unwinding involved in DNA replication
D0R2Q2	30S ribosomal protein S6	*rpsF*	9	12	255	−1.60	0.33	3.03	X		Translation
D0R268	Putative secreted protein	*FI9785_210*	21	102	323	−1.55	0.34	2.93		X	−
D0R3I4	Proline-tRNA ligase	*proS*	16	63	297	−1.38	0.39	2.60		X	Translation; tRNA aminoacylation for protein translation; prolyl tRNA aminoacylation; aminoacyl-tRNA metabolism involved in translational fidelity
D0R4U6	Valine-tRNA ligase	*valS*	20	101	323	−1.37	0.39	2.59	X		Translation; tRNA aminoacylation for protein translation; valyl tRNA aminoacylation; aminoacyl-tRNA metabolism involved in translational fidelity
D0R662	Phosphoenolpyruvate-dependent sugar phosphotransferase system EIIAB, probably mannose specific	*manL*	13	36	323	−1.16	0.45	2.24		X	Phosphoenolpyruvate-dependent sugar phosphotransferase system; carbohydrate transmembrane transport
D0R1P7	Muramidase	*FI9785_225*	8	64	323	−1.03	0.49	2.04		X	Metabolic process; peptidoglycan catabolic process; cell wall macromolecule catabolic process
D0R3J0	Translation initiation factor IF-2	*infB*	13	99	122	−1.02	0.49	2.03		X	Translation; translational initiation
D0R383	Methionine aminopeptidase	*pepM*	10	30	323	−0.96	0.52	1.94	X		Proteolysis; protein initiator methionine removal
D0R501	Probable transcriptional regulatory protein FI9785_1304	*FI9785_1304*	5	27	99	−0.88	0.54	1.84		X	Regulation of transcription, DNA-templated
D0R4I0	Pyruvate kinase	*pyk*	35	64	323	−0.88	0.54	1.84		X	Glycolytic process; phosphorylation
D0R395	Aminopeptidase	*pepN*	13	96	140	−0.87	0.55	1.83		X	Proteolysis
D0R2B3	50S ribosomal protein L10	*rplJ*	9	21	191	−0.85	0.56	1.80		X	Translation; ribosome biogenesis
D0R3F4	Oligopeptide-binding protein OppA	*oppA*	7	65	50	−0.81	0.57	1.76	X		Transmembrane transport
D0R1L8	Adenylate kinase	*adK*	13	24	323	−0.74	0.60	1.67		X	Nucleobase-containing compound metabolic process; nucleotide biosynthetic process; phosphorylation; AMP salvage; nucleoside monophosphate phosphorylation
D0R5L2	NH(3)-dependent NAD(+) synthetase	*nadE*	6	31	149	−0.71	0.61	1.63		X	NAD biosynthetic process

a WT, L. johnsonii FI9785; D, Δ*eps_cluster*; GO, gene ontology; –, no process identified.

b X, protein identified as having significantly different abundances between D and WT with this treatment.

One protein found at a lower level in the Δ*eps_cluster* strain—D0R1R2, encoded by *FI9785_242* (*242*; renamed *FI9785_RS00855*)—was identified using Rapid Annotations using Subsystems Technology (RAST) analysis as a bactoprenol glycosyltransferase, which is involved in cell wall biosynthesis. This was one of the three proteins identified by both iTRAQ and the label-free protocol. Blastp analysis indicated homology to the glycosyltransferase 2 superfamily, particularly to domains cd04187 (DPM1-like bac; expected value [E-value], 7.24e−81), the PRK10714 superfamily (undecaprenyl-phosphate 4-deoxy-4-formamido-l-arabinose transferase; E-value, 1.28e−33), pfam00535 (glycosyltransferase family 2; E-value, 6.63e−28), and COG0463 (glycosyltransferase involved in cell wall biosynthesis; E-value, 2.2e−26). This protein was selected for gene deletion to investigate a possible role in EPS1 biosynthesis.

### Deletion of *242* prevents biosynthesis of homopolysaccharide EPS1.

**FIG 3:**
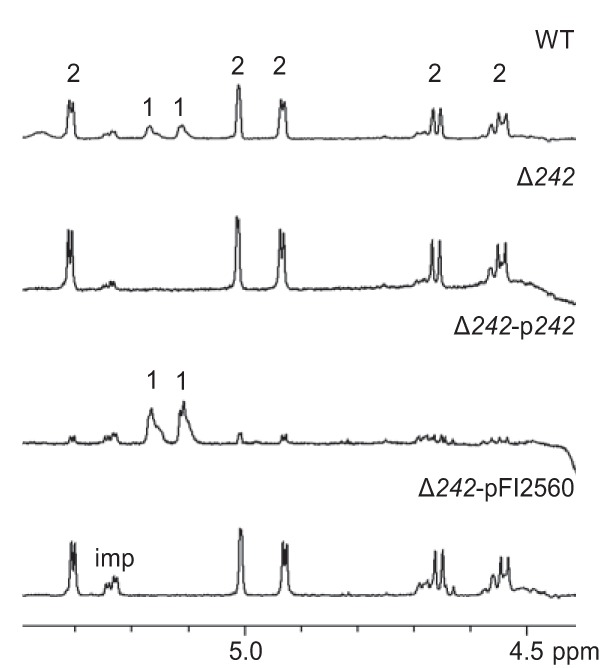
NMR analysis of pellet-associated EPS. 600 MHz ^1^H NMR spectra of EPS from WT and modified L. johnsonii (pellet samples, D_2_O, 338°K). Anomeric signals of EPS1 and EPS2 are labeled 1 and 2, respectively; imp, impurities. The presence of EPS1 is indicated by two H1 signals of equal intensity at 5.17 ppm [(1,2,6)-α-Glc] and 5.11 ppm (t-α-Glc). There are multiple H1 signals associated with EPS2 as indicated at the chemical shifts listed previously (20, 23).

The coding sequence for FI9785_242 (242) was deleted from the L. johnsonii FI9785 genome to create strain Δ*242*. Comparison of proton nuclear magnetic resonance (^1^H-NMR) profiles of EPS extracted from the WT and strain Δ*242* showed that EPS1 production was undetectable in samples extracted both from cell pellets and from supernatants (Fig. 3; Fig. S1A), indicating that 242 is essential for EPS1 production. NMR analysis of EPS extracted from a derivative of strain Δ*242* containing a plasmid expressing the *242* gene under the regulation of a strong constitutive promoter (Δ*242*-p*242*) showed that complementation restored EPS1 expression, with an increased ratio of EPS1 to EPS2 compared to the WT (Fig. 3; Fig. S1A).

Previous NMR analysis of EPS extracted from the Δ*eps_cluster* and Δ*epsA* strains and then purified by trichloroacetic acid (TCA) precipitation failed to detect EPS1 or EPS2 (20, 23). However, our analysis here of crude EPS preparations prior to TCA purification, using an increased temperature and higher number of scans, revealed the presence of EPS1 in both strains (Fig. S1B). This indicates that the genes in the *eps* cluster which produce EPS2 are not required for EPS1 production.

### 241-242 show homology to GtrA-GtrB and have homologues in Gram-positive bacteria.

**FIG 4:**
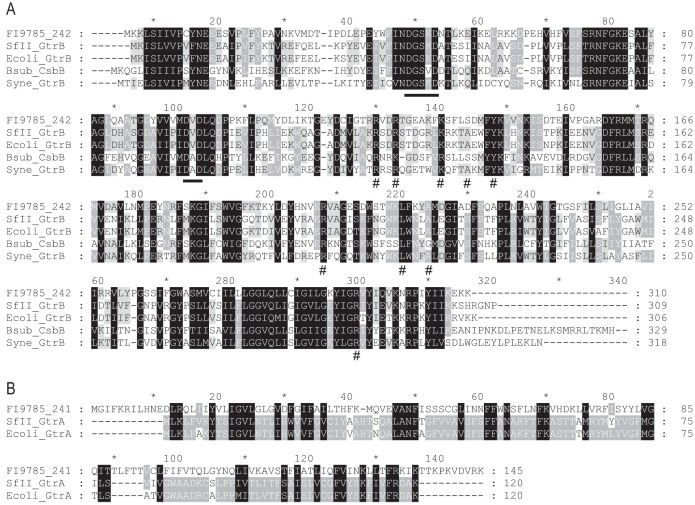
Amino acid alignments with GtrA and GtrB proteins. (A) Translation of *242* coding sequence (NCBI reference sequence WP_012845545) aligned with GtrB proteins from *Shigella* phage SfII (NCBI Protein accession number YP_008318506 [52]), E. coli K-12 (NCBI Protein accession number P77293 [53]), B. subtilis CsbB (NCBI Protein accession number Q45539 [54]) and *Synechocystis* sp. strains (NCBI Protein accession number Q55487 and Protein Data Bank number 5EKP [25]). Conserved motifs DXD and DXSXD are underlined, and residues affecting activity in 5EKP are marked with a #. (B) Translation of the *241* coding sequence (NCBI reference sequence WP_004896037) aligned with GtrA family proteins from *Shigella* phage SfII (NCBI Protein accession number YP_008318507 [52]) and E. coli K-12 (NCBI Protein accession number P77682 [53]). Black, dark gray, and light gray indicate 100%, 80%, and 60% homology, respectively.

Blastp analysis showed that amino acid homologues of 242 are widely distributed among *Lactobacillus* spp., with a high conservation of amino acid sequence (71 to 100% in the first 70 matches). Alignment of 242 with GtrB proteins from *Shigella* phage SfII and Escherichia coli, a putative bactoprenol glycosyltransferase CsbB from Bacillus subtilis, and a polyisoprenyl-phosphate glycosyltransferase from a *Synechocystis* sp. whose crystal structure has been solved (25) shows areas of homology across the whole sequence, including the motifs DXD and DXSXD, which have previously been identified as being conserved in glycosyltransferases (25–27) (Fig. 4A). Mutation of selected amino acids in the *Synechocystis* sp. GtrB was previously shown to affect enzymatic activity (25); all but one of these amino acids are conserved in 242 (Fig. 4A).

Blastp analysis of the translated product of the gene upstream of *242*, *FI9785_241* (*241*; renamed *FI9785_RS00850*), shows homology to domains pfam04138 (GtrA-like protein; E-value, 3.04e−18) and COG2246 (putative flippase GtrA; E-value, 2.78e−07). When aligned to the GtrA sequence pairing the SfII and E. coli GtrBs, 241 shows some conservation of sequence but less than that seen with the GtrB counterparts (Fig. 4B). GtrAB pairs have been identified in a range of Gram-negative bacteria and their bacteriophages and are commonly found with a glycosyltransferase GtrX, with the three-protein complex engineering the glycosylation of O-antigens with a single sugar moiety (28). However, we could not identify any further glycosyltransferases in the L. johnsonii FI9785 genome in the immediate vicinity of *241* and *242*.

**FIG 5:**
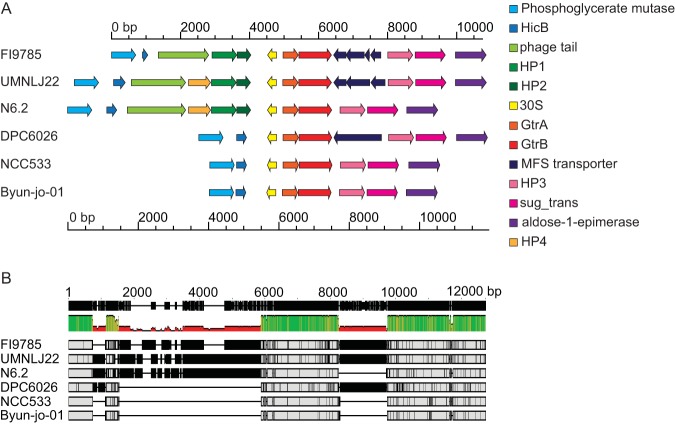
Conservation of genes with L. johnsonii strains from different environments. (A) ORFs are shown from genomes of L. johnsonii strains FI9785 (GenBank accession number FN298497 [49], nucleotides 184194 to 194938, loci *FI9785_RS00820* to *FI9785_RS00875*), UMNLJ22 (GenBank accession number NZ_CP021704 [T. J. Johnson and B. Youmans, unpublished], nucleotides 699996 to 711750, loci *A3P32_RS03290* to *A3P32_RS03350*); N6.2 (GenBank accession number NC_022909 [55], nucleotides 210473 to 221016, loci *T285_RS00860* to *T825_RS00915*); DPC6026 (GenBank accession number NC_017477 [56], nucleotides 202698 to 210932, loci *LJP_RS00920* to *LJP_RS00960*); NCC533 (GenBank accession number NC_005362 [57], nucleotides 196136 to 202659, loci *LJ_RS00845* to *LJ_RS00880*), and Byun-jo-01 (GenBank accession number NZ_CP029614 [D. Kim, unpublished], nucleotides shown in complement 1111505 to 1117990, loci *C0060_RS05265* to *C0060_RS05300*) with the GtrA-GtrB pairs aligned. (B) Nucleotide alignment of the sequences in panel A performed with Mauve to indicate areas of high sequence conservation. HicB, Hic B family antitoxin; phage tail, putative phage tail-related protein; HP, hypothetical protein; 30S, 30S ribosomal protein S14; MFS transporter, major facilitator family transporter; sug-trans, sugar transporter.

The *241-242* pair and surrounding genes show strong nucleotide conservation in other strains of L. johnsonii isolated from different sources. A surrounding 11.1-kb section encompassing 15 open reading frames (ORFs) from L. johnsonii FI9785 was compared with equivalent regions from annotated genomes of strains isolated from the human gut (NCC533), pig intestine (DPC6026), rat feces (N6.2), turkey (UMNLJ22), and mouse feces (Byun-jo-01), selecting the area between homologues of 2,3-diphosphoglycerate-dependent phosphoglycerate mutase and an aldose 1-epimerase family protein (Fig. 5). The conservation of ORFs surrounding the *gtrAB* pair varies among strains, with some ORFs being present but interrupted by stop codons. The section encoding the 30S ribosomal protein, *241* and *242*, is present in all genomes. Translated sequences of ORFs which are present in more than one genome show high amino acid similarity between strains; the 242 sequence (NCBI reference sequence WP_012845545) shows 99 to 100% identity with the equivalent sequences in the other genomes (NCBI reference sequences WP_012845545, WP_011161379, and WP_014567007). Alignment of the surrounding nucleotide region showed high conservation of the region covering the *241-242* pair, and analysis of these two genes in the 6 genomes showed between 97.1 and 99.8% nucleic acid identity with the FI9785 sequence (Fig. 5B). The central region of strong nucleotide conservation stretches from upstream of the 30S ribosomal gene to the noncoding sequence after *242*.

### *241* is required for EPS1 biosynthesis.

**FIG 6:**
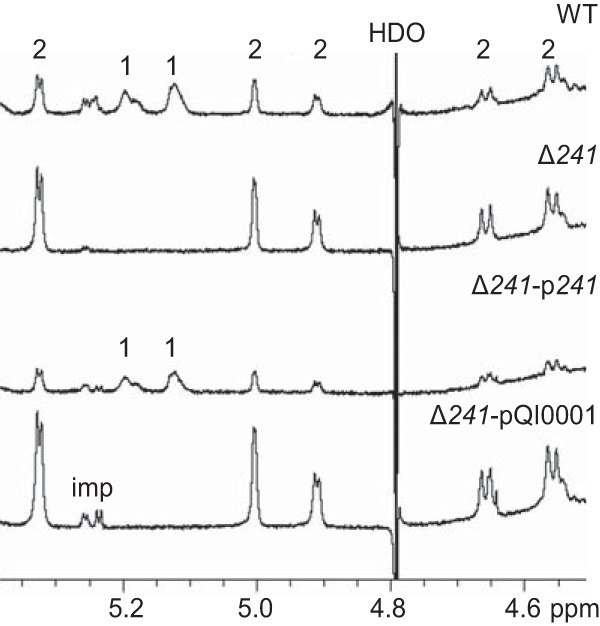
NMR analysis of pellet-associated EPS showing the effect of *241* deletion and complementation. The 600 MHz ^1^H NMR spectra of EPS from WT and engineered L. johnsonii (pellet samples, D_2_O, 300°K) are shown. Anomeric signals of EPS1 and EPS2 are labeled 1 and 2, respectively; imp, impurities.

To confirm the involvement of the putative flippase 241 in EPS1 production, a deletion mutant (Δ*241*) and its derivatives containing a *241* expression plasmid (Δ*241*-p*241*) or an empty plasmid control (Δ*241*-pQI0001) were constructed and their EPS analyzed using NMR. As with Δ*242*, gene deletion prevented EPS1 production while complementation restored biosynthesis (Fig. 6; Fig. S1C). A mutant where both *241* and *242* were deleted also showed production of EPS2 only (data not shown).

### EPS1 production affects growth.

**FIG 7:**
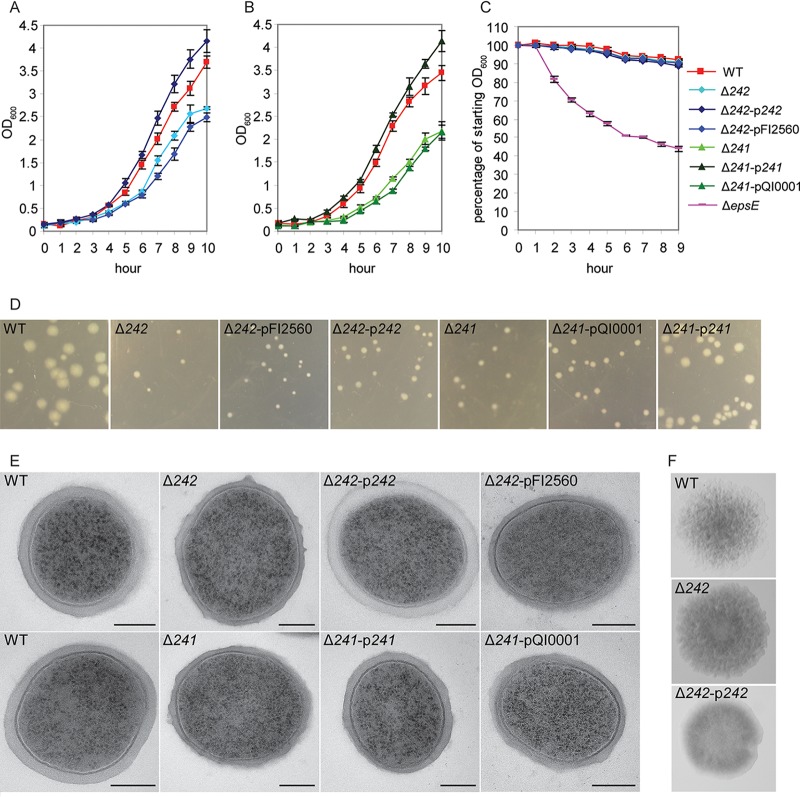
Phenotypic characterization of *241* and *242* deletion. (A and B) Growth of L. johnsonii strains in liquid at 37°C showing an increase in optical density. (C) Aggregation of overnight cultures. (D) Differences in colony size in strains given the same incubation time at 37°C. (E) TEM analysis of cells from overnight cultures (bar = 200 nm); WT, wild type. (F) Colony phenotypes.

The Δ*242* and Δ*241* strains both showed a slower growth phenotype than the wild type, both in liquid and on solid media (Fig. 7). This phenotype was similar when the strain contained an empty vector control, but normal growth was restored in liquid by overexpression of the *242* or *241* gene, although plate growth remained slightly retarded in the *242* complemented mutant. Mutant colonies did reach the size of typical 1-day WT colonies after further incubation within 2 days. The slow-growth phenotype was maintained during growth in anaerobic conditions and at a lower temperature (30°C). The presence or absence of EPS1 did not seem to affect aggregation, while as noted previously, nonproduction of EPS2 in Δ*epsE* caused a strong aggregation phenotype (21), suggesting that EPS2 is a primary contributor to low aggregation of the WT (Fig. 7C). Deletion of *242* also did not have a strong effect on colony phenotype, with colonies retaining a rough and crinkled appearance, although overexpression of *242* resulted in a smoother colony upon longer incubation. Transmission electron microscopy (TEM) showed that the Δ*242* and Δ*241* mutants retained a visible EPS layer; this was more frequently irregular than in WT samples (Fig. 7E). Cells overexpressing *242* or *241* also exhibited a thick EPS layer, and in the case of Δ*242*p*242*, this layer was consistently paler, suggesting a different response to the osmium staining.

## DISCUSSION

### Effect of EPS2 loss on the L. johnsonii FI9785 proteomic profile.

Apart from variations in proteins associated with ribosome structure, translation, and protein synthesis, very few biological processes seemed strongly affected in the soluble protein content by the loss of EPS2 synthesis in the Δ*eps_cluster* mutant strain. Comparative analysis of proteins from Lactobacillus plantarum grown at two temperature conditions, which gave a 10-fold difference in EPS production, also showed few changing proteins (29). It is interesting that loss of EPS2 production correlated with lower abundance of 242 in the Δ*eps_cluster* mutant strain than in the WT. We have now determined that this mutant is able to produce EPS1, but its biosynthesis is affected, either by the absence of the *eps* cluster genes or EPS2 itself or in response to changed cell conditions responding to reduction of a protective layer. The regulation of EPS synthesis has been linked to external signal and quorum sensing in a range of bacteria, including *L. plantarum* (30). BLAST analysis of a putative transcriptional regulator, D0R501, which was also less abundant in the Δ*eps_cluster* strain, showed a relationship to the YebC/PmpR family; regulators of this family are involved in a range of processes, including quorum sensing (31). Further investigation of the regulation of EPS1 and EPS2 genes, proteins, and polymers and how they relate to each other will be an interesting area for future study.

### Involvement of putative flippase and bactoprenol glycosyltransferase in homopolysaccharide biosynthesis in L. johnsonii.

The evidence from EPS NMR profiles from deletion and complementation strains indicates that putative bactoprenol glycosyltransferase 242 and neighboring putative flippase 241 are key components in the production of the branched glucan EPS1. In lactic acid bacteria, α-glucans such as dextran are commonly synthesized by glucansucrases, which cleave sucrose and then add glucose to a growing chain (17). Three other mechanisms of EPS and O-antigen polysaccharide (O-PS) biosynthesis have been described in bacteria—the Wzx/Wzy-dependent pathway, the ATP-binding cassette (ABC) transporter-dependent pathway, and the synthase-dependent pathway (32). The first two mechanisms begin with the addition of a phosphorylated monosaccharide from a UDP-sugar to a lipid carrier, commonly thought to be undecaprenyl phosphate (5, 33, 34), while the synthase pathway utilizes cytosolic nucleotide-activated sugars (35, 36). Guan and coworkers described a three-gene operon, *gtrABX*, involved in O-antigen glycosylation in a bacteriophage infecting Shigella flexnerii and demonstrated that bactoprenol glucose transferase GtrB transferred [^14^C]glucose to decaprenyl phosphate *in vitro* (28). They proposed a model where GtrB catalyzes the transfer of glucose from UDP-glucose to bactoprenol, GtrA flips the complex across the cytoplasmic membrane, and specific glycosyltransferase GtrX transfers the glucose to a specific residue on the O-antigen repeating unit (28). More recently, GtrB homologues have been shown to be involved in glycosylation of lipoteichoic and wall teichoic acids, and a similar 3-component mechanism has been proposed (37–39).

Our hypothesis is that 242 acts as a GtrB homologue, adding a glucose molecule to a lipid carrier, while the product of neighboring gene *241* functions as a flippase. However, the full process of chain and branch formation, and the possible involvement of glycosyltransferases elsewhere in the genome, remains to be determined. *241-242* may be involved in the decoration of a linear chain synthesized by other enzymes or may be an integral part of a biosynthetic cluster. The ability of bacterial glycosyltransferases to act on different substrates and even in different pathways has been noted (40). The genes encoding the three-component system involved in Staphylococcus aureus lipoteichoic acid glycosylation are not all located together on the chromosome (38), so it would not be unprecedented for a distant gene(s) to be involved in a three- or four-component EPS biosynthetic pathway. The genome of L. johnsonii FI9785 contains several other glycosyltransferase genes which may be involved in synthesis of a linear chain, acting in concert with 241-242 to produce the final external EPS1. It is hoped that further examination of these genes will lead to a clearer model for the synthesis of this unusual EPS.

### Effect of *242* or *241* deletion on L. johnsonii.

Mutations affecting L. johnsonii FI9785 EPS synthesis have been shown to affect aggregation, biofilm formation, adhesion to human HT29 cells and chicken gut explants, and resistance to stress, suggesting that EPS has a protective capacity (20, 21, 23, 41). We found that gene deletion of *242* or *241* slowed bacterial growth. The slow-growth phenotype is still seen at lowered temperatures or in the absence of oxygen, suggesting that it is not caused by increased sensitivity of cells to these conditions due to a reduction of the EPS layer. Further, removal of EPS2 did not seem to have the same effect, as the Δ*eps_cluster* mutant strain showed a similar growth rate to the wild type when grown for proteomic analysis. It has been noted that mutations which might prevent the release of undecaprenyl phosphate by blocking the full EPS biosynthetic process affect cell viability, either by reducing the amount of undecaprenyl available for other processes or by membrane destabilization in the presence of lipid intermediates (5, 42). However, it is not obvious why deletion of a protein proposed to glycosylate the lipid carrier might have a similar effect unless there are other components of EPS1 biosynthesis that might also interact with the carrier.

In conclusion, we found that a putative glycosyltransferase, 242, was less abundant in the Δ*eps_cluster* strain and that deletion of its gene prevented the accumulation of EPS1, while plasmid complementation restored production. *In silico* analysis indicated that *242* and its preceding gene, *241*, show similarity to two members of a three-component system, *gtrABX*, shown to mediate O-antigen glycosylation in Gram-negative bacteria and, more recently, to be involved in teichoic acid glycosylation in Gram-positive species. Further deletion and complementation studies showed that *241* was also essential for EPS1 production. High conservation of nucleotide sequence with other L. johnsonii strains and the presence of analogous genes in other lactobacilli suggest that this might be part of a novel mechanism for homopolysaccharide EPS biosynthesis in Gram-positive bacteria. EPS/O-PS biosynthetic pathways have been studied in detail, but many questions remain unanswered, and new enzymes are still being discovered (43). Given the potential technological applications of EPS, there is significant interest in engineering novel forms (32), and their important roles in protection and biofilm formation make EPS biosynthesis a valid target for novel strategies to control pathogens. Further discovery of alternative mechanisms may give future opportunities to both understand and exploit bacterial EPS synthesis.

## MATERIALS AND METHODS

### Bacterial strains and growth conditions.

L. johnsonii strains were grown as described previously (41) in homemade De Man Rogosa Sharpe medium (MRS) using 2% glucose as a carbon source at 37°C. Lactococcus lactis MG1614 (44) was grown in GM17 (Oxoid) at 30°C. Plasmids were selected and maintained using chloramphenicol (pFI2560 and pQI0001) at 7.5 μg ml^−1^ or 5 μg ml^−1^ and erythromycin (pG^+^host9) at 10 μg ml^−1^ and 5 μg ml^−1^ for L. johnsonii and L. lactis, respectively. The L. johnsonii strains and plasmids produced and/or used in this study are listed in Table 2.

**TABLE 2:** L. johnsonii strains created and used in this study

Strain	Genotype	Description	Plasmid	Reference
FI9785	Wild type	Poultry isolate		18
FI10754	Δ*eps_cluster*	*eps* gene cluster deleted		20
FI11504	Δ*242*	FI9785 with *242* gene deleted		This study
FI11646	Δ*242*-p*242*	FI11504 complemented with the *242* gene in expression plasmid pFI2560	pFI2843	This study
FI11647	Δ*242*-pFI2560	FI11504 with pFI2560 empty vector control	pFI2560	This study
FI11669	Δ*241*	FI9785 with *241* gene deleted		This study
FI11670	Δ*241*-p*241*	FI11669 complemented with the *241* gene in expression plasmid pQI0001	pQI0002	This study
FI11671	Δ*241*-pQI0001	FI11669 with pQI0001^*a*^ empty vector control	pQI0001^*a*^	This study
FI10785	Δ*epsA*	*epsA* transcriptional regulator from *eps* gene cluster deleted		23
FI10844	Δ*epsE*	*epsE* priming glycosyltransferase from *eps* gene cluster deleted		21

a Plasmid pFI2560 with cloning site NcoI altered to NdeI-BamHI.

### Isolation of proteins.

Soluble protein extracts were prepared from L. johnsonii FI9785 and Δ*eps_cluster* strains inoculated from overnight cultures at 2% into prewarmed medium and grown to an optical density (OD_600_) of 2.0 (6 to 7 h). Cells from 15-ml aliquots were harvested by centrifugation at 3,000 × *g* for 15 min at 4°C, washed with 5 ml phosphate-buffered saline (PBS) containing 1× cOmplete protease inhibitor (Roche), recentrifuged, washed with 1 ml PBS/protease inhibitor, and recentrifuged at 13,000 × *g* for 2 min at 4°C before removal of the supernatant and freezing on dry ice. Three biological replicates and one technical replicate were prepared for each strain. Pellets were resuspended in 500 μl extraction buffer (50 mM HEPES [pH 7.7], 0.3% SDS, 1× protease inhibitor, 5 U ml^−1^ RNase-free DNase [Promega], 10 mM MgSO_4_, and 1 mM CaCl_2_) and then sonicated using a Soniprep 150 (Sanyo) for 7 cycles of 15 s with 30 s incubation on ice between cycles. After centrifugation at 13,000 × *g* for 25 min at 4°C to pellet debris, the supernatant was precipitated overnight with 5 volumes of cold acetone at –20°C. Proteins were collected by centrifugation at 14,000 × *g* for 10 min at 4°C and stored at –20°C. Total soluble protein was resuspended in 250 μl 0.5 M trimethylammonium bicarbonate buffer (Sigma), 0.05% SDS, and 1× protease inhibitor and stored in LoBind tubes (Eppendorf). Concentrations were measured using Bradford reagent (Bioline).

### Quantitative mass spectrometry.

Bacterial protein samples, three biological replicates of mutants and controls and one technical replicate, were digested by trypsin, and the tryptic peptides were labeled using the iTRAQ 8-plex kit (AB Sciex Pte. Ltd., USA) following the manufacturer’s instructions. The samples of each experiment were pooled and fractionated using a high-pH reversed-phase peptide fractionation kit (Pierce, Thermo Fisher Scientific). Each single fraction was analyzed using an nLC MS/MS Orbitrap Fusion trihybrid mass spectrometer coupled with a nano flow ultrahigh-performance liquid chromatography (UHPLC) system (Thermo Fisher Scientific). The peptides were separated after being trapped on a C_18_ precolumn, using a gradient of 3 to 40% acetonitrile in 0.1% formic acid over 50 min at a flow rate of 300 nl min^−1^ at 40°C. The peptides were fragmented in the linear ion trap by a data-dependent acquisition method, selecting the 40 most intense ions. For label-free experiments, each tryptic peptide sample was analyzed in triplicate as described above. All analyses were performed in triplicate. The raw data were analyzed with MaxQuant version 1.6.2.3 (Resource Identification Portal [RRID]:SCR_014485) using Andromeda software and consulting the Uniprot_ Lactobacillus johnsonii (strain FI9785) (1,726 sequences) protein database; the tolerance on parents was 10 ppm and on fragments was 0.02 ppm. The variable modifications allowed were oxidation on methionine and carboxyamidomethylation on cysteine as fixed modifications. The false discovery rate was below 1% using a decoy and reverse database, and the identified proteins contained at least 2 peptides with at least 6 amino acids sequenced. iTRAQ and label-free quantitative analyses were also performed using MaxQuant software and evaluated using Perseus statistical software (RRID:SCR_015753) with a two-sided *t* test, setting a *P* value of less than 0.05 and false-discovery rate (FDR) less than 0.01. Gene Ontology analyses were performed using the QuickGO algorithm European Molecular Biology Laboratory, European Bioinformatics Institute (EMBL-EBI; RRID:SCR_004608).

### Plasmid construction and gene deletion.

Genes were deleted from the L. johnsonii FI9785 chromosome as described previously (21) using the thermosensitive vector pG+host9 (45) containing a knockout cassette of the partial upstream and downstream genes, amplified and joined by splice overlap extension PCR using primers designed to generate restriction sites for cloning and to create spliced products (see supplemental text and Table 3). Initial cloning was performed using electrocompetent Lactococcus lactis MG1614 (46) with growth at 28°C. After sequence confirmation, plasmids were transformed into electrocompetent L. johnsonii FI9785 (47), and gene replacement was performed as described previously (45) using 30°C as the permissive temperature and 42°C as the nonpermissive temperature. For recovery of Δ*242* and Δ*241*, it was necessary to recover deletions at 30°C, and excised plasmids were cured by successive subculturing. For complementation, the *242* gene was cloned into the L. johnsonii expression plasmid pFI2560 (21), and the *241* gene was cloned into pFI2560-derivative pQI0001 (see supplemental text); the ligation products and control vector were transformed into electrocompetent L. johnsonii FI9785 as before.

**TABLE 3:** Oligonucleotide primers used for creation of deletion constructs and plasmids and assessment of sequence integrity, integration, and excision

Primer	Sequence 5′–3′^*a*^
241Eco_F	GAT**G**AATTCACGCTGCTTAG
241splice243_R	**CGGCTTTTTGTCATAT**ACTTTAACAGTCTTTCTTAT
243Spe_R	CT**AC**TAGTCATGATTGATTTTGGT
243splice241_F	**AGAAAGACTGTTAAAGT**ATATGACAAAAAGCCGA
241_IF	GCTTCTACGTCACCAGCTTCT
243_IR	TCCACAGTTTCGAACTGGTG
240_F	ATGTCTAAAGTGTGACTATATGTT
240splice242_R	**TACTTTAACAGTCTTTCTTA**GGCTTATTTTCCCTTCT
242splice240_F	**AGAAGGGAAAATAAGCC**TAAGAAAGACTGTTAAAGTA
242Spe_R	CATTTGAC**T**AGTCATCATTCGGTAGTC
240_IF	GAATGTCTAAAGTGTGACTATATGTT
242_IR	ACGGTTGTATTCAGGCATATTC
pGhost1	AGTCACGACGTTGTAAAACGACG
pGhostR	TACTACTGACAGCTTCCAAGG
pForVec	ACAGCAATGTTACAAGTTGAAAT
p181	GCGAAGATAACAGTGACTCTA
242_COD2F	AAAAAATTATCAATTATAGTTCCTTG
242_C_R	GAAGCTCCACGTGAACTTC
241_NdeF	TAA**CATA**TGGGTATTTTTAAAAGAATAC
241_BamR	TTT**GG**AT**C**CTTTAACAGTCTTTCTTATTAC

a Mismatching base pairs to insert restriction sites or for splice overlap extension are in bold.

### Bioinformatic analysis.

Translated gene sequence homologies and domain searches were performed using Blastp (RRID:SCR_001010) (48). The L. johnsonii genome FN298497 (49) was reanalyzed using RAST (RRID:SCR_014606) (50). Amino acid alignments were performed using the clustalW algorithm (RRID:SCR_002909) in Vector NTI (Invitrogen; RRID:SCR_014265) and visualized using GeneDoc. Nucleotide alignments were performed using Geneious (Biomatters Ltd., New Zealand; RRID:SCR_010519); short sequences were aligned using Geneious alignment, and larger genome segments were aligned using Mauve (RRID:SCR_012852) (51).

### Isolation and NMR spectroscopy of EPS.

Crude EPS was isolated from 2-day 500-ml cultures grown in MRS at 37°C as described previously (21), except that the initial extraction of capsular EPS from the washed bacterial pellet was performed by sonication in 50 ml 1 M NaCl for 7 cycles of 45 s with 30 s incubation on ice between cycles, followed by centrifugation at 6,000 × *g* and 4°C for 30 min to remove bacterial debris before the rounds of ethanol precipitation, the initial ethanol precipitation was for 3 days instead of overnight, and crude EPS was not further purified by TCA precipitation. EPS samples were analyzed using NMR as before (20) but with heating to 338°K (Δ*242* series) and an increased number of scans (1,024). Samples in the Δ*241* series were measured at 300°K.

### Growth, aggregation, and phenotype studies.

Overnight (15-h) cultures of WT, Δ*epsE*, Δ*242*-p*242*, and Δ*241*-p*241* strains and 20-h cultures of Δ*242*, Δ*242*-pFI2560, Δ*241*, and Δ*241*-pQI0001 strains were used as inocula for growth and aggregation studies. For liquid growth, 20-ml broths were inoculated at 2%, and the OD_600_ of 10-fold diluted samples was measured every hour during aerobic growth at 37°C. Colony size on plates was monitored aerobically at 30°C and 37°C and anaerobically at 37°C. All liquid growth of plasmid-containing strains was supplemented with chloramphenicol, while plate growth was nonselective. For aggregation, triplicate 1-ml samples from vortexed overnight cultures were transferred to cuvettes, and the OD_600_ was measured hourly during incubation at room temperature. Growth and aggregation assays were each performed three times, and representative curves are shown. TEM images were taken from overnight cultures as described previously (20).

### Data availability.

All strains reported in this work are deposited in the Quadram Institute Bioscience culture collection and are available from the corresponding author upon request. For accession numbers, see Table 2.

## Supplementary Material

Supplemental file 1

Supplemental file 2
