# Supplementary material for: Identification of Genes Required for Glucan Exopolysaccharide Production in Lactobacillus johnsonii Suggests a Novel Biosynthesis Mechanism
Source: Appl Environ Microbiol. 2020 Apr 1;86(8):e02808-19. doi: 10.1128/AEM.02808-19 (PMC7117936; doi:10.1128/AEM.02808-19)
Supplement: Supplemental file 1 [file AEM.02808-19-s0001.pdf]

Supplemental Text - PCR amplification and subcloning

PCR for creation of deletion constructs and plasmids was performed with Phusion (Finnzymes) with dNTPs from Bioline and oligonucleotide primers, listed in Table 3, from Sigma Genosys, using 5 ng *L. johnsonii* FI9785 genomic DNA as a template and the conditions recommended by the manufacturer. Genomic DNA was extracted from mid exponential phase cells using Genomic Tip 20/G columns and genomic DNA buffer set (Qiagen), with the addition of 50 U mutanolysin (Sigma) to aid cell lysis. Annealing temperatures were 5 cycles with the nearest neighbour (NN) of the sequence of the primer with 100% match to the template, calculated using OligoCalc (Kibbe, WA., Nuc Ac Res 35:W43-46, 2007, doi:10.1093/nar/gkm234), and 20 cycles at the temperature calculated with the Finnzyme Phusion calculator using the whole primer. For splice overlap, annealing temperatures used the NN of the overlap region, or the Phusion calculation of the outer primers if lower. All purifications used Sureclean (Bioline).

To make the deletion construct for *242*, primary splice products made with 241Eco_F and 241splice243_R (partial *241* with EcoRI site and overhang to match *243* – 707 bp) and 243splice241_F and 243Spe_R (partial *243* with SpeI site and overhang to match *241* – 656 bp) were spliced and amplified with 241Eco_F and 243Spe_R (*241-243* – 1330 bp). To make the deletion construct for *241*, primary splice products were made with 240_F and 240splice242_R (partial *240* upstream of SpeI site and overhang to match *242* – 521 bp) and 242splice240_F and 242Spe_R (partial *242* with SpeI site and overhang to match *240*– 537 bp), and these were spliced and amplified with 240_F and 242Spe_R (*240-242* – 1021 bp). Spliced PCR products were digested with the appropriate restriction enzymes (New England Biolabs) and ligated into restricted pG+host9 vector, which had been dephosphorylated with Antarctic phosphatase (NEB), using Fast-Link DNA ligase (Epicentre Biotechnologies). Plasmids were prepared using the EZNA plasmid kit (Omega) with an initial 15 min incubation at 37°C with 5 mg ml^{-1} lysozyme and 3 U mutanolysin and sequencing was performed by Eurofins. Transformants, integrants and deletions were confirmed by colony PCR using GoTaq G2 polymerase (Promega). pGhost1 and pGhostR gave a product of 1494 bp with pG+host9-*241-243* and 1181 bp from pG+host9-*240-242*. After integration of the *242* deletion construct, pGhost1 and 243_IR gave a product of 2395 bp, indicating insertion in *241*. After excision, 241_IF and 243_IR gave 1393 bp for a deletion and 2326 bp for the wild type genotype. After integration of the *241* deletion construct, pGhost1 and 242_IR gave a product of 1547 bp, indicating insertion in *240.* After excision, 240_IF and 242_IR gave 1099 bp for a deletion and 1497 bp for the wild type.

For complementation, the *242* gene was amplified from *L. johnsonii* genomic DNA with Phusion from the second codon using 242_COD2F and 242_C_R. The 964 bp product was phosphorylated with T4 polynucleotide kinase and ligated to *L. johnsonii* expression plasmid pFI2560 which had been

restricted with NcoI, end-filled with T4 DNA polymerase and dephosphorylated with Antarctic phosphatase. All enzymes were from New England Biolabs and used as recommended. Primers surrounding the cloning site, pForVec and p181, were used to confirm the presence of the gene (1155 bp). For complementation of *241* the gene was amplified from genomic DNA with primers 241_NdeF and 241_BamR, restricted and cloned into restricted dephosphorylated pQI0001 (a derivative of vector pFI2560 where the NcoI site was replaced with NdeI/BamHI sites (Mayer, unpublished)). Primers pForVec and p181 gave a product of 639 bp.

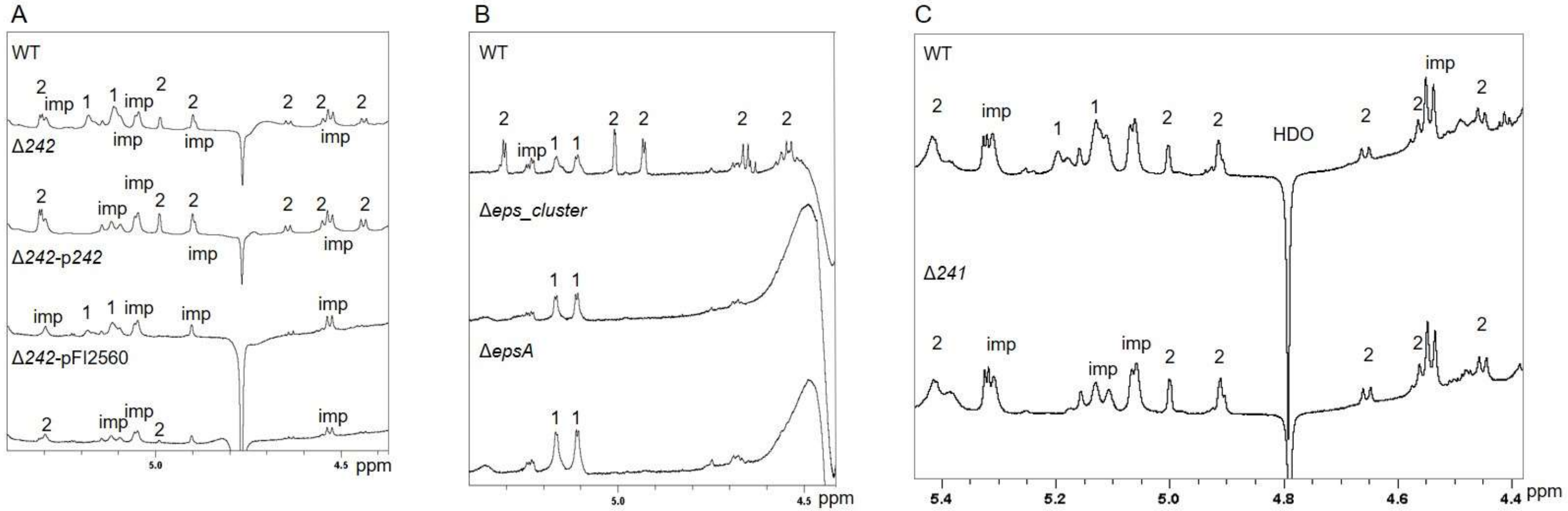

Supplementary Figure S1 A, NMR analysis of crude extracts of cell culture supernatants from the same experiments that provided the pellet-associated EPS in Fig. 3. NMR spectra were run in the same way as in Fig, 3 except that the temperature was 300^{0}K. Spectra show the same combinations of EPS (labelled '1' and '2') as in the corresponding samples in Fig. 3 but the level of impurities from the medium (imp) is greater. In particular, EPS1 is completely absent from Δ*242*. This was confirmed by running a TOCSY spectrum of Δ*242* (data not shown) which showed that none of the impurity peaks in the vicinity of the two EPS1 H1 signals were associated with EPS1. Previous work showed these impurity peaks were found in control samples not inoculated with bacteria (Dertli E, Colqhoun IJ, Gunning AP, Bongaerts RJ, Le Gall G, Bonev BB, Mayer MJ and Narbad A, J Biol Chem 288:31938-51, 2013, doi:10.1074/jbc.M113.507418). **B,** 600 MHz spectra of *L. johnsonii* WT and Δ*eps_cluster* and Δ*epsA* mutants (crude pellet samples, D_2O, 338^{0}K). EPS1 is present in the mutants but not EPS2. **C,** NMR analysis of crude extracts of supernatants from the same experiments that provided the pellet-associated EPS from WT and Δ*241* samples in Fig. 6.
